# RPARP‐AS1 Targets miR‐10b‐5p to Influence Prognosis in Non‐Small Cell Lung Cancer and Inhibit Tumor Progression

**DOI:** 10.1155/ancp/8817867

**Published:** 2026-10-06

**Authors:** Conghui Wang, Jing Zhang, Xin Wang, Jianzhu Yang, Zhenlong Zhu, Ruoshi Yuan, Duojun Qiu

**Affiliations:** ^1^ Department of Pathology The First Hospital of Hebei Medical University Shijiazhuang 050023 China hebmu.edu.cn

**Keywords:** miR-10b-5p, non-small cell lung cancer, prognosis, progression, RPARP-AS1

## Abstract

**Objective:**

To investigate the roles of RPARP‐AS1 and miR‐10b‐5p in regulating the prognosis and tumor progression of non‐small cell lung cancer (NSCLC).

**Methods:**

A total of 138 NSCLC patients were enrolled. Surgical specimens were stored in liquid nitrogen. All patients received 5 years of clinical follow‐up for prognostic assessment. In addition, multivariate Cox regression analysis was conducted to identify independent factors affecting NSCLC prognosis, and gene expression levels were quantified by reverse transcription quantitative polymerase chain reaction (RT‐qPCR). Cell Counting Kit‐8 (CCK‐8) assays were performed to examine cell proliferation, and transwell assays were used to assess cell migration and invasion. Furthermore, nuclear‐cytoplasmic fractionation experiments were conducted to localize RPARP‐AS1 distribution. The interaction between RPARP‐AS1 and miR‐10b‐5p was examined by dual luciferase reporter assays, and downstream targets in NSCLC were predicted using databases.

**Results:**

RPARP‐AS1 was downregulated, while miR‐10b‐5p was upregulated in both NSCLC patient tissues and cells. Patients with reduced RPARP‐AS1 expression or increased miR‐10b‐5p expression had lower survival rates, accompanied by worsened tumor size, differentiation, and metastasis. oe‐RPARP‐AS1 inhibited tumor cell proliferation, migration, and invasion. However, RPARP‐AS1‐induced functional alterations in NSCLC cells were mediated by miR‐10b‐5p. A total of 47 overlapping genes were identified to potentially mediate downstream signaling pathways of the RPARP‐AS1/miR‐10b‐5p axis in NSCLC cells.

**Conclusion:**

Silencing RPARP‐AS1 targeted miR‐10b‐5p to induce NSCLC cell proliferation, migration, and invasion, leading to tumor progression and poor prognosis.

## 1. Introduction

Non‐small cell lung cancer (NSCLC) constitutes a relatively common lung tumor, primarily covering adenocarcinoma, squamous cell carcinoma, and large‐cell lung cancer [1]. The 5‐year survival rate for NSCLC patients remains at merely 10%. Compared with other lung cancer subtypes, NSCLC displays relatively slow proliferation and delayed metastatic progression [2]. Many patients are diagnosed at an intermediate or advanced stage, resulting in a poor prognosis. As one of the treatment options for early‐stage NSCLC, surgical resection presents a relatively high recurrence rate [3]. Meanwhile, chemoradiotherapy regimens for advanced patients are associated with significant adverse reactions [4]. Recent studies demonstrate targeted therapy and immune checkpoint therapy as emerging treatment modalities [5–7]. For example, studies reveal the remarkable efficacy of PEGylated liposomal gefitinib in treating NSCLC [8], extending patient survival to some extent. Novel immunotherapies such as a dual PD‐1/VEGF blockade show superior efficacy compared to pembrolizumab. This opens novel avenues for NSCLC treatment. Furthermore, research on applying 3D deep learning and radiological techniques to early NSCLC diagnosis [9] holds promise for improving early detection rates and patient prognosis. However, a comprehensive analysis of extensive literature reveals the following unresolved controversies concerning early NSCLC diagnosis and treatment: First, for early‐stage NSCLC patients undergoing local therapy, the widespread adoption of single‐fraction stereotactic radiotherapy remains limited [10, 11]. Second, for advanced patients, limitations such as tumor heterogeneity, scarcity of therapeutic targets, and side effects associated with novel therapies hinder their treatment efficacy [12, 13]. Regarding prognostic assessment, academic consensus remains elusive on the predictive value of certain biomarkers. Therefore, identifying reliable biomarkers to monitor NSCLC progression and predict patient prognosis, alongside dissecting their regulatory mechanisms in tumor cells, holds considerable significance for developing innovative therapeutic approaches against NSCLC.

lncRNAs cannot be translated into proteins, but they play a significant role in regulating cellular processes and disease development [14, 15]. RPARP‐AS1 (also known as C10orf95‐AS1 and C10orf95 antisense RNA 1) is located on human chromosome 10q24.32, primarily distributed in the endoplasmic reticulum, nucleus, and plasma membrane. Accumulating evidence has revealed the context‐dependent roles of RPARP‐AS1 in human cancers. For instance, it acts as a protective factor against endoplasmic reticulum stress in lung adenocarcinoma (LA) [16] while being significantly downregulated in NSCLC patients, correlating with poor overall survival (OS) [17]. Additionally, studies have reported that RPARP‐AS1 plays a role in the immune microenvironment of lung cancer, which is associated with prognosis [18]. However, its regulatory role in the prognosis and tumor progression of NSCLC patients remains poorly understood. Previous laboratory studies have identified binding sites between miR‐10b‐5p and RPARP‐AS1. Studies have shown that miR‐10b‐5p is overexpressed in NSCLC [19] and LA patients [20]. Elevated miR‐10b‐5 levels are independently associated with OS in NSCLC patients [21]. miR‐10b‐5 may be a potential prognostic marker for LA [22]. Additionally, studies have also confirmed miR‐10b‐5p’s involvement in the inflammatory cytokine storm induced by severe acute respiratory syndrome coronavirus (SARS‐CoV) [23, 24]. To this end, it is hereby speculated that RPARP‐AS1 may mediate the regulatory role of miR‐10b‐5p in NSCLC patients.

Based on the above background, 138 NSCLC patients were enrolled, and their 5‐year postoperative prognosis was tracked. On the one hand, by analyzing the relationship between patient characteristics and target gene expression in NSCLC patients, this study revealed its potential clinical value as a prognostic biomarker and a therapeutic target. On the other hand, the molecular mechanisms of lncRNA and miRNA involvement in NSCLC were further validated through gene functional experiments. Collectively, this study may provide novel biomarkers for the early diagnosis and prognosis assessment of NSCLC, offering theoretical support for developing low‐toxicity, highly effective targeted therapeutic drugs.

## 2. Materials and Methods

### 2.1. Case Information

The significance level (*α*) was set at 0.05, test power (1‐*β*) at 0.8, estimated effect size at 0.4, and dropout rate at 20% using 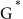
Power software (two‐tailed test). This calculation indicated a minimum sample size of 125 cases. Among the initial 233 lung cancer patients enrolled, 95 suspected cases were confirmed as other lung cancer types (small‐cell lung cancer, lung cancer metastases, etc.) or pulmonary diseases were thus excluded. Ultimately, 138 patients were diagnosed with NSCLC through rigorous screening and pathological examination. All patients were hospitalized and treated at The First Hospital of Hebei Medical University between April 2018 and March 2019. None of them reported other severe pulmonary diseases that could affect NSCLC studies (e.g., chronic obstructive pulmonary disease, tuberculosis, and pulmonary fibrosis,). Postoperative tumor tissue and adjacent normal tissue (< 2 cm) were frozen at −80°C and then stored in liquid nitrogen. These patients were followed up for 5 years. This study was approved by the Ethics Committee of the First Hospital of Hebei Medical University, and all patients and their families signed written informed consent forms. Inclusion criteria were as follows: (1) adult patients; (2) newly diagnosed, with no prior treatment history (including radiotherapy, chemotherapy, or drug therapy); (3) patient tumor node metastasis (TNM) stage classified as stage I–IV; and (4) no severe abnormalities in bone marrow reserve function, cardiac function, or liver and kidney function. Those with a history of drug allergies, mental confusion preventing cooperation, or severe organ failure were excluded from this study.

### 2.2. Data Analysis

Initially, the potential downstream target genes of miR‐10b‐5p were predicted using the TargetScan database (https://www.targetscan.org/vert_80/), miRDB database (https://mirdb.org/), and miRWalk database (http://mirwalk.umm.uni-heidelberg.de/). Concurrently, all potential genes involved in NSCLC were retrieved from Genecard. Common genes from the three databases were intersected with those predicted from Genecard to obtain overlapping genes. Subsequently, the overlapping genes were input into the STRING website (https://cn.string-db.org/) for protein–protein interaction (PPI) construction. Hub genes were identified by scoring and ranking the output results. Additionally, the Gene Ontology (GO) and Kyoto Encyclopedia of Genes and Genomes (KEGG) information of the overlapping genes was enriched using the Micro‐Bioinformatics website (http://www.bioinformatics.com.cn/plot_basic_gopathway_enrichment_bubbleplot_081). Besides, the subcellular localization of RPARP‐AS1 was predicted using two independent web‐based bioinformatics tools, iLoc‐lncRNA 2.0 (http://lin-group.cn/server/iLoc-LncRNA(2.0)/predictor.php) and lncLocator (http://www.csbio.sjtu.edu.cn/bioinf/lncLocator/). The RPARP‐AS1 sequence was uploaded to both servers according to their respective protocols, with predicted subcellular compartments and corresponding probability scores accordingly being recorded.

### 2.3. Cell Culture

Beas‐2B (Cat: CBP60577) represents a normal lung epithelial cell line, while A549 (Cat: CBP60084), HCC1195 (Cat: CBP61199), H1975 (Cat: CBP60121), and NCI‐H460 (Cat: CBP60138) are NSCLC cell lines, all obtained from the Chinese Cell Bank. All cell lines were delivered with fewer than five in‐house passages in strict accordance with the manufacturer’s guidelines. These cells exhibited low passage numbers, excellent condition, and high viability. Furthermore, all cells underwent STR typing (see Table S1) and contamination monitoring (negative for HIV‐1, HBV, HCV, mycoplasma, bacteria, and fungi). Upon receipt, cells were transferred from T25 flasks to 10 cm culture dishes using trypsin (Gibco, USA). Culture conditions were maintained at 37°C with 5% CO_2_. Passaging was performed when the cell density reached 70% or higher. After two rounds of passaging, a portion of the cells was cryopreserved, and the rest were reserved for subsequent experiments. A549 cells were cultured using Kaighn’s modified Ham’s F‐12 (N6658, Sigma–Aldrich, Germany) nutrient mixture, while Beas‐2B cells were cultured using DMEM/F‐12 medium (DMEM: Ham’s F‐12 = 1:1, Gibco, USA). HCC1195, H1975, and NCI‐H460 cells were cultured using a RPMI‐1640 medium (Gibco, USA). Additionally, the basal medium was supplemented with 10% fetal bovine serum (Gibco, USA) and 1% penicillin/streptomycin (P/S, Gibco, USA).

### 2.4. Cell Transfection

Cell transfection was performed using the EZ Trans Cell Transfection Reagent (AC04L092, Life‐iLab, Shanghai). One day before transfection, the cells were transferred to a 24‐well plate for growth. Transfection was performed when the cell density reached 70%–80%, and the amount per well in the 24‐well plate was calculated. Then, 1 μg of plasmid DNA (oe‐NC and oe‐RPARP‐AS1) or oligonucleotide (mimic NC and miR mimic) was diluted with 40 μL of serum‐free basal medium, and 3 μL of EZ Trans transfection reagent was diluted with 40 μL of basic medium. Following that, the diluted transfection reagent was added to the diluted plasmid DNA/oligonucleotide, mixed thoroughly, and incubated at room temperature for 10 min. 80 μL of the mixture was incorporated into each well and mixed gently. The cells were incubated in a constant‐temperature incubator for 12 h.

### 2.5. Reverse Transcription Quantitative Polymerase Chain Reaction (RT‐qPCR)

Before the experiment, the tissue must be ground using the Tissue Master high‐throughput tissue grinder (E6618) from Beyotime. RNA was extracted from the NSCLC tissue and cells using the Ultrapure RNA Kit (CW0581, Cwbiotech, Taizhou), and miRNA was obtained using the miRcute miRNA isolation Kit (DP501, TIANGEN BIOTECH, Beijing). The quality of the extracted RNA was assessed using a Nanodrop 8000 microvolume nucleic acid quantifier. Besides, reverse transcription of RPARP‐AS1 and miR‐10b‐5p was performed using the HiFiScript cDNA Synthesis Kit (CW2569, Cwbiotech, Taizhou) and the PrimeScript RT Reagent Kit (RR037, TAKARA, Japan), respectively. Ultimately, RT‐qPCR reactions for lncRNA and miRNA were separately conducted with the Ultra SYBR Mixture (CW0957, Cwbiotech, Taizhou) and miRNA qPCR Assay Kit (CW2142, Cwbiotech, Taizhou), with the specific conditions set as follows: 95°C for 10 min, 95°C for 15 s, 60°C for 1 min, and repeated 42 times. Raw data were recorded using an ABI 7500 Fluorescent Quantitative PCR Instrument and normalized using the 2^−ΔΔCt^ method. For data normalization, β‐actin and U6 were employed as internal reference genes for RPARP‐AS1 and miR‐10b‐5p, respectively. The corresponding primer sequences were as follows: RPARP‐AS1, forward (F), 5^′^—GTCTGTCTACACCTGGGCTGGAG—3^′^, and reverse (R), 5^′^—GGGAGAATCGCTTGAACCTGGAAG—3^′^. β‐actin, *F*, 5^′^—CACTGAGCGAGGCTACAGCTT—3^′^; *R*, 5^′^—TTGATGTCGCGCACGATTT—3^′^. miR‐10b‐5p, *F*, 5^′^—GGGTACCCTGTAGAACCG—3^′^, *R*, 5^′^—AACTGGTGTCGTGGAGTCGGC—3^′^. U6, 5^′^—CTCGCTTCGGCAGCACA—3^′^; *R*, 5^′^—AACGCTTCACGAATTTGCGT—3^′^.

### 2.6. Cell Counting Kit‐8 (CCK‐8) Assay

Cell proliferation capacity was assessed using the CCK‐8 (AC11L054, Life‐iLab, Shanghai). Posttransfection cells were trypsin‐digested and centrifuged at 1000 × *g* for 5 min. Harvested cell pellets were resuspended in a complete medium. A 100 μL aliquot (~1 × 10^3^ cells) was seeded into each well of a 96‐well plate, followed by 24 h of incubation in a culture incubator. At 0, 24, 48, and 72 h, 10 μL of CCK‐8 solution was added to the corresponding wells, and incubation was continued in a constant‐temperature cell culture incubator for 2 h. Finally, the absorbance at 450 nm was measured using a multi‐mode microplate reader.

### 2.7. Transwell Assay

The transfected cells were transferred to transwell chambers, replaced with serum‐free medium, and starved for 24 h. For the invasion assay, a layer of matrix gel must be spread on the bottom of the upper chamber of the transwell chamber. After cell seeding, the chambers were incubated in a constant‐temperature incubator. The cell culture plate was removed, and 500 μL of cell culture medium containing 10% fetal bovine serum was added to the lower chamber. Cells were continuously cultured at 37°C and 5% CO_2_ for 12 h. Excess medium was discarded, and the upper transwell chambers were rinsed with PBS before fixation in formaldehyde fixative (P0099, Beyotime, Shanghai) for 5 min, which was then permeated in 80 μL of methanol (≥99.8%, 322415, Sigma–Aldrich, Germany) for 15 min and finally stained with Giemsa (C0133, Beyotime, Shanghai). PBS rinses were performed after each step. Finally, the average value of five random fields of view under the microscope was selected as the number of cells migrating or invading in that well.

### 2.8. Nuclear‐Cytoplasmic Fractionation Assay

Nuclear‐cytoplasmic fractionation assay was carried out using the PARIS kit (AM1921, Life Technologies, Carlsbad, CA, USA) to isolate cytoplasmic and nuclear RNA. In brief, 5 × 10^6^ cells were gently resuspended in the kit’s lysis buffer and kept on ice for 10 min for complete lysis. The lysate was centrifuged at 11,000 rpm for 10 min at 4°C, after which the supernatant (cytoplasmic fraction) was carefully transferred to a new tube for RNA purification. The remaining nuclear pellet was resuspended in an ice‐cold cell disruption buffer to release nuclear RNA. Following the manufacturer’s protocol, RNA from both fractions was eluted using the kit’s elution buffer. RPARP‐AS1 distribution between the cytoplasm and nucleus was determined by RT‐qPCR, with GAPDH serving as the cytoplasmic endogenous control and U6 as the nuclear endogenous control.

### 2.9. Dual Luciferase Reporter Assay

Wild‐type (WT‐RPARP: 5^′^—UUUUUUGUAGAGACAGGGUU—3^′^) and mutant (MUT‐RPARP: 5^′^—UUUAAGCUAGAGUGUCCCAU—3^′^) plasmids were constructed using the pmirGLO vector. The target plasmid, control plasmid, and oligonucleotides (miR‐10b‐5p mimic/inhibitor) were transfected into cells using the EZ Trans transfection reagent. Subsequent assays were performed using the Renilla‐Firefly Luciferase Dual Assay Kit (HY‐K1013, MCE, USA). Following transfection, the culture medium was removed, and the cells were washed with PBS. A total of 50 μL of 1× lysis buffer was added to each well of a 48‐well plate and incubated at room temperature for 15 min. Then, the lysate was transferred to a new centrifuge tube and centrifuged at 4°C and 12,800 rpm for storage. A total of 20 μL of the cell lysate was added to a black microplate. First, 100 μL of firefly luciferase working solution was incorporated, and the firefly luciferase signal was measured using a microplate reader. Subsequently, 100 μL of sea squirt luciferase working solution was added, and the sea squirt luciferase signal was detected, with the experimental results expressed as the relative luminescence values obtained from the ratio of the two signals.

### 2.10. Result Analysis

Effects of target gene expression on the pathological characteristics of NSCLC patients were analyzed using IBM SPSS Statistics 25.0, and GraphPad Prism 9.0 was employed to compare the differences between experimental data. All experiments were performed in triplicate. Intergroup comparisons were performed using *t*‐tests and analysis of variance (ANOVA). Multivariate Cox regression was used to identify independent factors influencing NSCLC patient mortality. Kaplan–Meier (KM) survival curves were employed to record patient prognosis over 5 years. Additionally, the relationship between RPARP‐AS1 and miR‐10b‐5p was assessed via Pearson correlation analysis, with  ^∗^ *p*  < 0.05 indicating statistically significant differences in data results.

## 3. Results

### 3.1. Changes in RPARP‐AS1 and miR‐10b‐5p Expression in NSCLC Patients and Cells

Results revealed significantly downregulated RPARP‐AS1 levels in NSCLC tumor tissues compared to adjacent normal tissues (*p* < 0.0001, *d*_Cohen_ = 1.09, Figure 1A). Additionally, RPARP‐AS1 expression was observed to be more noticeably downregulated in NSCLC patients with more advanced TNM staging (*p* = 0.0001, Figure 1B). Cell experiment results showed obviously downregulated RPARP‐AS1 in all four NSCLC cell lines (*p* = 0.0442, Figure 1C). Additionally, it was found that miR‐10b‐5p was markedly upregulated in tumor tissues from NSCLC patients (*p*  < 0.0001, *d*_Cohen_ = 1.19, Figure 1D). Furthermore, the upregulation of miR‐10b‐5p increased with the progression of NSCLC TNM staging (*p*  < 0.0001, Figure 1E). miR‐10b‐5p expression was remarkably reduced in NSCLC cells compared to Beas‐2B cells (*p* = 0.0335, Figure 1F).

**Figure 1:**
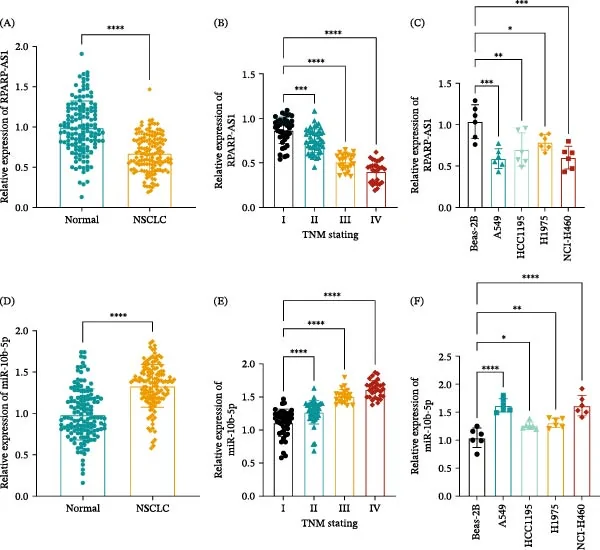
Expression of RPARP‐AS1 and miR‐10b‐5p and their relationship with pathological characteristics in NSCLC patients. (A) Expression of RPARP‐AS1 in NSCLC and adjacent normal tissue. (B) Expression levels of RPARP‐AS1 in NSCLC patients with different TNM stages. (C) Expression changes of RPARP‐AS1 in human normal lung epithelial cells and lung cancer cells. (D) Expression of miR‐10b‐5p in NSCLC and adjacent normal tissue. (E) Expression of miR‐10b‐5p in patients with different TNM stages. (F) Expression changes of miR‐10b‐5p in human normal lung epithelial cells and lung cancer cells. Data are mean ± SD from three biological replicates (*n* = 3, each with 6 technical replicates).  ^∗^ *p* < 0.05,  ^∗∗^ *p*  < 0.01,  ^∗∗∗^ *p*  < 0.001,  ^∗∗∗∗^ *p*  < 0.0001 vs. normal or TNM staging I, or Beas‐2B.

### 3.2. Impact of RPARP‐AS1 and miR‐10b‐5p Expression on Pathological Characteristics and Prognosis in NSCLC Patients

Based on the above experimental results, NSCLC patients were further grouped according to the average expression levels of RPARP‐AS1 and miR‐10b‐5p in their tissue samples. As presented in Table 1, patients with low RPARP‐AS1 expression exhibited a greater incidence of tumors ≥4 cm (*p* = 0.004), poor differentiation, lymph node metastasis, and TNM stage III–IV disease (*p*  < 0.0001). However, no apparent differences were identified between the two groups in terms of age, gender, smoking history, or drinking history. Additionally, as shown in Table 2, NSCLC patients with enhanced miR‐10b‐5p expression had a higher prevalence of smoking (*p* = 0.038), larger tumors (*p* = 0.002), more advanced disease (*p* = 0.034), metastasis, and more severe TNM staging (*p*  < 0.0001). However, miR‐10b‐5p expression exerted no impact on age, gender, and alcohol consumption history. Prognostic analysis revealed that patients with downregulated RPARP‐AS1 and upregulated miR‐10b‐5p were more likely to experience death (*p*  < 0.0001, Figure 2A,C,). Furthermore, the present experimental results indicated that higher RPARP‐AS1 and lower miR‐10b‐5p expression levels were associated with improved survival rates in NSCLC patients (*p*  < 0.0001, Figure 2B,D,). Furthermore, multivariate Cox analysis (Table 3) identified low RPARP‐AS1 expression (*p* = 0.043) and high miR‐10b‐5p levels (*p* = 0.034) as independent risk factors associated with poor survival in NSCLC patients.

**Figure 2:**
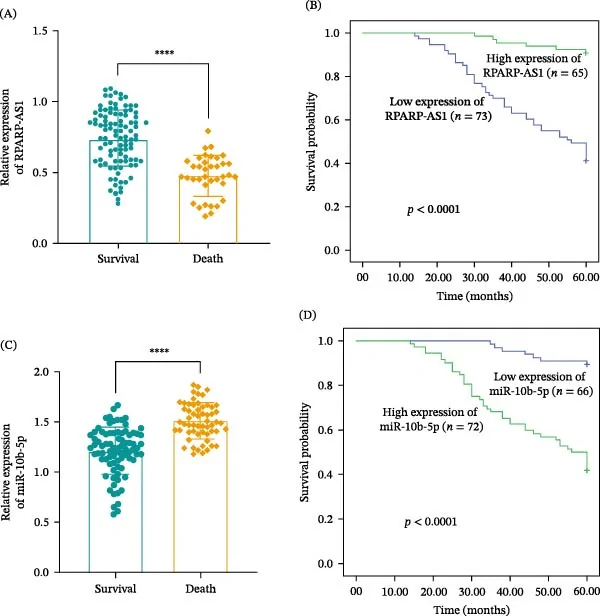
The impact of RPARP‐AS1 and miR‐10b‐5p expression on the prognosis of NSCLC. (A) RPARP‐AS1 expression in survivors and deceased patients. (B) KM curve analysis of survival in patients with different RPARP‐AS1 expression levels. (C) miR‐10b‐5p expression in the tissues of survivors and deceased patients. (D) KM curve analysis of survival outcomes in patients with different miR‐10b‐5p expression levels. Data are mean ± SD from three biological replicates (*n* = 3, each with 6 technical replicates).  ^∗∗∗∗^ *p*  < 0.0001 vs. survival.

**Table 1:** Effect of RPARP‐AS1 expression on pathological characteristics of NSCLC.

Characteristics	RPARP‐AS1 expression		*p*
	Low (*n* = 73)	High (*n* = 65)	
Age (years)	61.78 ± 11.27	62.57 ± 10.29	0.669
Gender, *n* (%)	—	—	0.163
Female	42 (57.53)	45 (69.23)	—
Man	31 (42.47)	20 (30.77)	—
Smoking, *n* (%)	—	—	0.057
No	37 (50.68)	44 (67.69)	—
Yes	36 (49.32)	21 (32.31)	—
Drinking, *n* (%)	—	—	0.110
No	43 (58.90)	47 (72.31)	—
Yes	30 (41.10)	18 (27.69)	—
Tumor size, *n* (%)	—	—	0.004
＜4 cm	41 (56.16)	52 (80.00)	—
≥4 cm	32 (43.84)	13 (20.00)	—
Differentiation, *n* (%)	—	—	＜0.0001
None/low	36 (49.32)	51 (78.46)	—
Medium/high	37 (50.68)	14 (21.54)	—
Lymph node metastases, *n* (%)	—	—	＜0.0001
No	32 (43.84)	51 (78.46)	—
Yes	41 (56.16)	14 (21.54)	—
TNM staging, *n* (%)	—	—	＜0.0001
Ⅰ–Ⅱ	35 (47.95)	52 (80.00)	—
Ⅲ–Ⅳ	38 (52.05)	13 (20.00)	—

*Note:* *p*  < 0.05 indicated that there was a significant difference in the data results.

Abbreviation: TNM, tumor node metastasis.

**Table 2:** Relationship between miR‐10b‐5p expression and pathological characteristics of NSCLC patients.

Characteristics	miR‐10b‐5p expression		*p*
	Low (*n* = 66)	High (*n* = 72)	
Age (years)	62.39 ± 10.61	61.93 ± 11.01	0.802
Gender, *n* (%)	—	—	0.077
Female	47 (71.21)	40 (55.56)	—
Man	19 (28.79)	32 (44.44)	—
Smoking, *n* (%)	—	—	0.038
No	45 (68.18)	36 (50.00)	—
Yes	21 (31.82)	36 (50.00)	—
Drinking, *n* (%)	—	—	0.210
No	47 (71.21)	43 (59.72)	—
Yes	19 (28.79)	29 (40.28)	—
Tumor size, *n* (%)	—	—	0.002
＜4 cm	53 (80.30)	40 (55.56)	—
≥4 cm	13 (19.70)	32 (44.44)	—
Differentiation, *n* (%)	—	—	0.034
None/low	48 (72.73)	39 (54.17)	—
Medium/high	18 (27.27)	33 (45.83)	—
Lymph node metastases, *n* (%)	—	—	＜0.0001
No	53 (80.30)	30 (441.67)	—
Yes	13 (19.70)	42 (58.33)	—
TNM staging, *n* (%)	—	—	＜0.0001
Ⅰ–Ⅱ	52 (78.79)	35 (48.61)	—
Ⅲ–Ⅳ	14 (21.21)	37 (51.39)	—

*Note:* *p*  < 0.05 indicated that there was a significant difference in the data results.

Abbreviation: TNM, tumor node metastasis.

**Table 3:** Cox regression analysis predicts independent factors affecting NSCLC patients.

Factors	*p*	HR	95% CI
RPARP‐AS1	0.043	0.368	0.140–0.969
miR‐10b‐5p	0.034	2.689	1.078–6.709
Smoking	0.050	1.784	0.999–3.185
Drinking	0.113	1.603	0.895–2.872
Tumor size	0.058	1.791	0.982–3.269
Differentiation	0.073	1.781	0.947–3.351
Lymph node metastases	0.058	1.875	0.979–3.589
TNM staging	0.053	1.807	0.993–3.291

*Note*: *p*  < 0.05 indicated that there was a significant difference in the data results.

Abbreviation: TNM, tumor node metastasis.

### 3.3. RPARP‐AS1 Overexpression Leading to Functional Changes in NSCLC Cells

When oe‐RPARP‐AS1 was transfected into NSCLC cells, RPARP‐AS1 was noticeably upregulated in both A549 and NCI‐H460 cells (*p* = 0.0001, Figure 3A). Cell functional assays revealed that overexpression of RPARP‐AS1 in NSCLC cells obviously inhibited cell proliferation (*p*  < 0.0001, Figure 3B). Additionally, oe‐RPARP‐AS1 transfection was observed to inhibit the migration (*p* = 0.0003, Figure 3C) and invasion (*p* = 0.0122, Figure 3C) activities of NSCLC cells.

**Figure 3:**
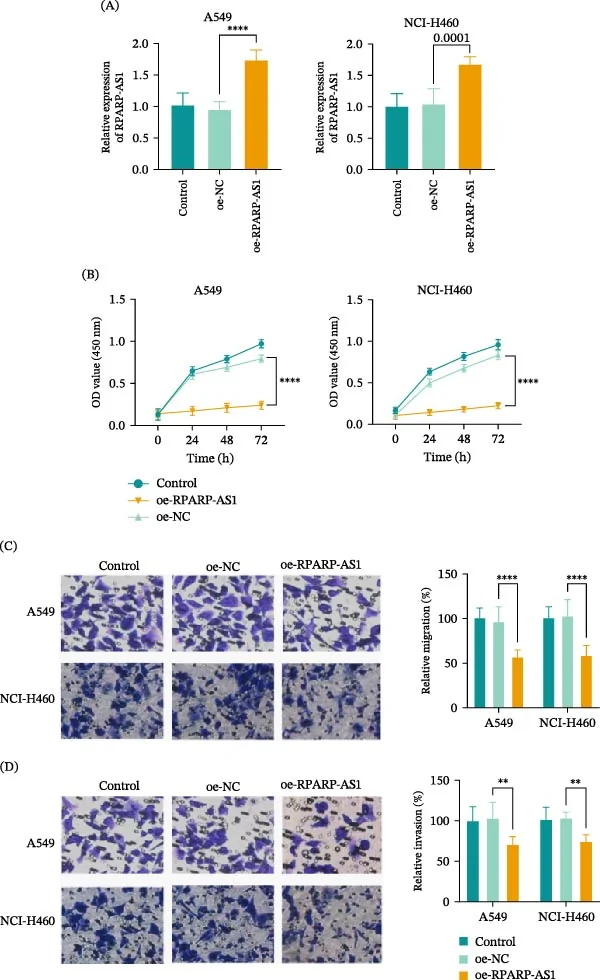
Effects of RPARP‐AS1 overexpression on NSCLC cell function. (A) Construction of RPARP‐AS1 overexpression vectors. (B) Effects of oe‐RPARP‐AS1 transfection on the proliferation of A549 and NCI‐H460 cells. (C) Effects of RPARP‐AS1 overexpression on the migration of two NSCLC cell lines. (D) Effects of RPARP‐AS1 overexpression on the invasion of NSCLC cells. Data are mean ± SD from three biological replicates (*n* = 3, each with 6 technical replicates).  ^∗^ *p*  < 0.05,  ^∗∗^ *p*  < 0.01,  ^∗∗∗^ *p*  < 0.001,  ^∗∗∗∗^ *p*  < 0.0001. vs. control and oe‐NC.

### 3.4. miR‐10b‐5p as a Target Gene of RPARP‐AS1 in Regulating NSCLC Cells

Analysis results from the StarBase database (https://rnasysu.com/encori/) indicated that miR‐10b‐5p had a target site with RPARP‐AS1, with the binding sequences of RPARP‐AS1 and miR‐10b‐5p shown in Figure 4A. A nuclear‐cytoplasmic fractionation assay suggested that RPARP‐AS1 was primarily located in the cytoplasm (Figure 4B). This finding was further supported by prediction analyses using iLoc‐lncRNA 2.0 and the LncLocator. They both indicated that RRARP‐AS1 was predominantly distributed in the cytoplasmic compartment, especially in the cytosol (Figure S1), consistent with the argument of its role as a molecular sponge to adsorb target miRNAs. When cells containing the wild‐type plasmid were co‐transfected with miR‐10b‐5p mimic, the luciferase activity was obviously reduced, whereas the opposite trend was observed when the transfection adopted miR‐10b‐5p inhibitor (*p*  < 0.05, Figure 4C). Additionally, this study identified a significant negative correlation between RPARP‐AS1 and miR‐10b‐5p expression in NSCLC tissues (*r* = −0.7825, *p*  < 0.0001, Figure 4D).

**Figure 4:**
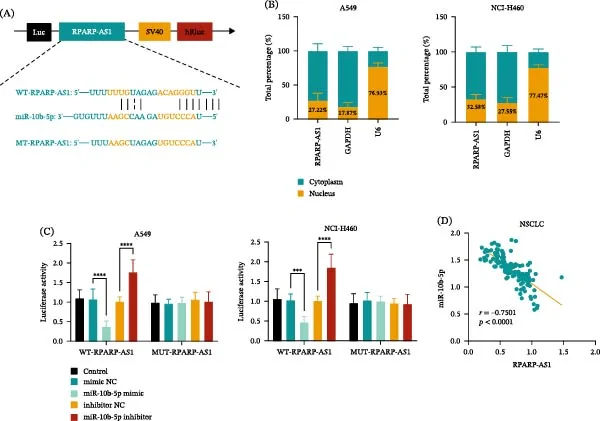
miR‐10b‐5p is a target gene of RPARP‐AS1. (A) Sequence of the miR‐10b‐5p target site in RPARP‐AS1. (B) Nuclear‐cytoplasmic fractionation assay of RPARP‐AS1. (C) Dual luciferase reporter assay validating the target relationship between RPARP‐AS1 and miR‐10b‐5p. (D) Correlation between RPARP‐AS1 and miR‐10b‐5p expression in tumor tissue from NSCLC patients. Data are mean ± SD from three biological replicates (*n* = 3, each with 6 technical replicates).  ^∗∗∗^ *p*  < 0.001,  ^∗∗∗∗^ *p*  < 0.0001 vs. mimic NC or inhibitor NC.

### 3.5. RPARP‐AS1 Relies on miR‐10b‐5p to Inhibit NSCLC Cell Function

Transfection of oe‐RPARP‐AS1 markedly inhibited the miR‐10b‐5p expression (*p* = 0.0004). However, when miR‐10b‐5p mimic was transfected into cells, the expression level of miR‐10b‐5p increased (*p* = 0.0355, Figure 5A). Furthermore, the results showed that the inhibition of cell proliferation caused by RPARP‐AS1 overexpression was rescued by the miR‐10b‐5p mimic (*p*  < 0.0001, Figure 5B). Additionally, miR‐10b‐5p overexpression was observed to counteract the inhibition of cell migration (*p* = 0.0099, Figure 5C) and invasion (*p* = 0.0335, Figure 5D) arising from oe‐RPARP‐AS1.

**Figure 5:**
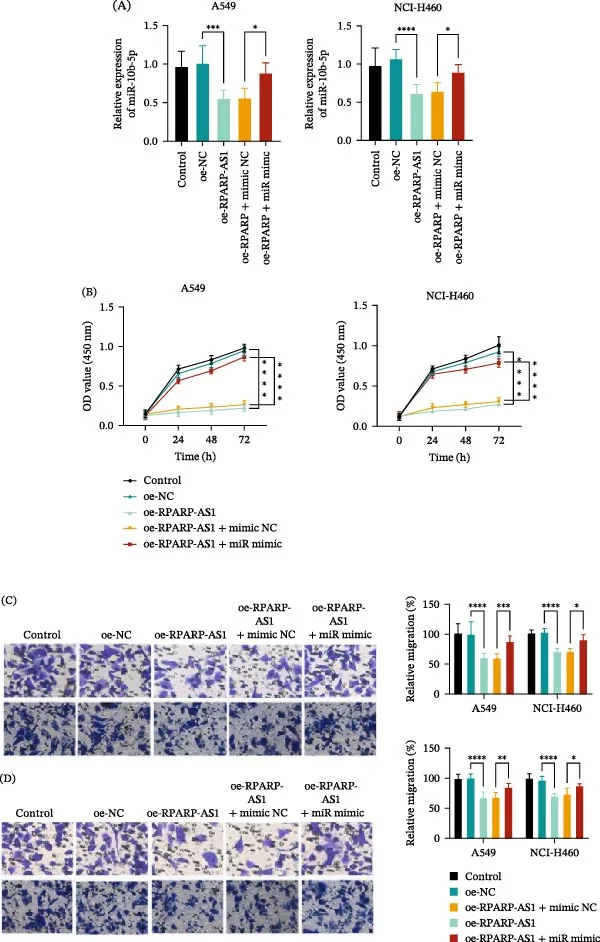
RPARP‐AS1 targets miR‐10b‐5p to regulate NSCLC cell function. (A) Levels of miR‐10b‐5p in NSCLC cells after overexpression of RPARP‐AS1/miR‐10b‐5p. (B) Effect of transfecting miR‐10b‐5p mimic on the proliferation capacity of NSCLC cells. (C) Changes in migration of NSCLC cells after overexpression of miR‐10b‐5p. (D) Effect of overexpression of miR‐10b‐5p on the invasion of NSCLC cells. Data are mean ± SD from three biological replicates (*n* = 3, each with 6 technical replicates).  ^∗^ *p*  < 0.05,  ^∗∗^ *p* < 0.01,  ^∗∗∗^ *p*  < 0.001,  ^∗∗∗∗^ *p*  < 0.0001. vs. control and oe‐NC and oe‐RPARP‐AS1 + mimic NC.

### 3.6. Analysis of Downstream Target Genes and Potential Signaling Pathways of miR‐10b‐5p

To elucidate the mechanisms of RPARP‐AS1 targeting miR‐10b‐5p in NSCLC, the potential target genes of miR‐10b‐5p were predicted using bioinformatics tools. The TargetScan, miRDB, and miRWalk databases yielded a total of 101 overlapping genes. However, when genes involved in the NSCLC process from Genecard were intersected with the aforementioned results, 47 overlapping genes were identified (Figure 6A). Subsequently, a PPI was constructed using the STRING website. The results revealed a network with 47 nodes and 36 edges. Based on the scoring results, 10 overlapping genes, including CDK6, CREB1, NCOR2, ANK1, BCR, SDC1, TFRC, ACTG1, CRK, and ITGB8, were involved in more events related to NSCLC (Figure 6B). GO functional analysis results (Figure 6C) demonstrated that the overlapping genes were mainly enriched in biological processes (BPs), including axonogenesis, cellular response to glucose stimulus, cellular response to nerve growth factor stimulus, response to nerve growth factor, and ephrin receptor signaling pathway. The cellular components (CCs) of the overlapping genes were primarily located in the transcription regulator complex, axon development, the cytoplasmic side of the plasma membrane, cell–cell junctions, and histone methyltransferase complexes. The molecular functions (MFs) of these genes included focal adhesions, cAMP‐binding, DNA‐binding transcription activator activity, RNA polymerase activity, and binding to signal receptor complex docking activity. KEGG signal enrichment results indicated the possible involvement of the 47 overlapping genes in cell adhesion factor activity, chronic myeloid leukemia, longevity regulation pathways, proteoglycan formation in cancer cells, hypertrophic cardiomyopathy, the PI3K‐Akt signaling pathway, and actin cytoskeleton regulation (Figure 6D).

**Figure 6:**
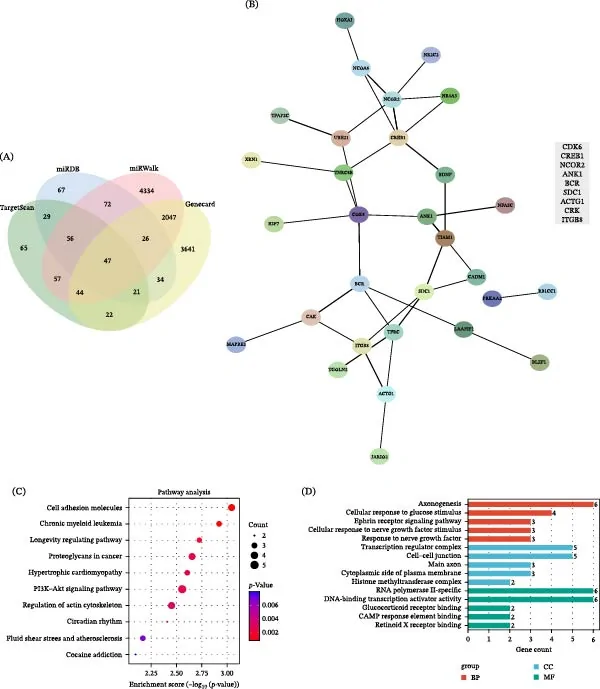
Prediction of target genes and signaling pathways downstream of miR‐10b‐5p involved in NSCLC regulation. (A) Target genes downstream of miR‐10b‐5p involved in NSCLC regulation predicted by four different databases. (B) Construction of a protein–protein interaction (PPI) network for the 47 overlapping genes. (C) GO functional analysis results for the 47 overlapping genes. (D) KEGG pathway enrichment results for the 47 overlapping genes.

## 4. Discussion

An increasing number of studies have reported the role of lncRNA in NSCLC. Specifically, [25] Lin et al. [17] found that seven lncRNA features could predict OS in NSCLC. Miao et al. [26] observed the efficacy of lncRNA in predicting the prognosis risk in elderly NSCLC patients. Herein, the results revealed that downregulation of RPARP‐AS1 or increased expression of miR‐10b‐5p was associated with severe tumor staging in NSCLC patients. Patients with low RPARP‐AS1 expression exhibited poorer prognosis outcomes, while elevated miR‐10b‐5p levels corresponded to decreased 5‐year survival. Multiple studies have found that lncRNA typically targets miRNAs to participate in NSCLC progression. For example, lncRNA MRPL39 inhibits NSCLC cell proliferation and migration through the miR‐30/TSC1 axis, exerting an antitumor effect [27]. Knockdown of LINC01296 inhibits NSCLC via the miR‐143‐3 p/ATG2B axis [28]. LINC00472 targets the miR‐1275/HOXA2 axis to inhibit the malignant progression of NSCLC. Herein, the results [29] demonstrated that RPARP‐AS1 overexpression effectively inhibited NSCLC cell proliferation, migration, and invasion. However, transfection with the miR‐10b‐5p mimic counteracted the effects of RPARP‐AS1 upregulation on the cellular function. Additionally, low RPARP‐AS1 expression was observed to be associated with poor survival in breast cancer [30], and RPARP‐AS1 was also found to correlate with prognosis in colorectal cancer [31] and endometrial cancer [32].

Studies have shown that various targeted or immunotherapy methods have been tested in the treatment of NSCLC [33]. lncRNAs in plasma exosomes can serve as predictive factors for immunotherapy [34]. Nicolescu et al. [35] regulated the oncogenic lncRNA DANCR for NSCLC treatment using ECO/siRNA nanoparticles. Rana et al. [36] reported sialic acid‐chitosan nanoparticles as a beneficial method for treating NSCLC. Researchers achieved tumor regression in xenografted mice through the targeted action of CLA‐coated paclitaxel‐superparamagnetic iron oxide nanoparticles [37]. Additionally, Yuan et al. [38] identified lysine methyltransferase 5C (KMT5C) as a key factor in NSCLC immune evasion, and targeting KMT5C was found to enhance immune checkpoint blockade. Ni et al. [39] reported immune checkpoints as one of the key strategies for improving stage IV NSCLC. Moreover, studies have shown RPARP‐AS1 as a characteristic of N‐6‐methylated LA, capable of influencing the tumor microenvironment [40], rendering targeted therapy targeting RPARP‐AS1 and immune checkpoint inhibition an important direction for future NSCLC treatment.

Upregulation of miR‐10b‐5p has been reported to promote necrotic apoptosis in NSCLC [41]. However, this study made the first attempt to report miR‐10b‐5p’s involvement in NSCLC regulation and its regulation by RPARP‐AS1. Furthermore, it successfully demonstrated that the functional promotion of NSCLC cells by RPARP‐AS1 downregulation was also achieved through miR‐10b‐5p. Studies have shown that miR‐10b‐5p is upregulated in extracellular vesicles from systemic sclerosis patients [42]. Elevated levels of miR‐10b‐5p have also been detected in the cerebrospinal fluid from dogs with brain tumors [43]. For instance, Zhou et al. [44] found that miR‐10b‐5p may serve as a diagnostic biomarker for colorectal cancer. Abnormal expression of miR‐10b‐5p has been observed in epilepsy associated with glioma [45]. However, when analyzing the functional roles of the top 10 hub genes among 47 overlapping genes, the present study found that they appeared to participate in the regulation of lung cancer through different pathways. Additionally, enrichment analysis revealed that overlapping genes were primarily enriched in processes such as cell–cell junctions, axon development, and the cAMP or PI3K/Akt signaling pathways. For example, ANK1 and ITGB8 are major components of focal adhesions. CDK6, CREB1, and BCR act as activators of the cAMP pathway during neural development. SDC1, TFRC, and ACTG1 are proteins associated with plasma membrane integrity or movement. Previous studies have shown that certain components of intercellular junctions (e.g., integrins and E‐cadherin) are associated with the differentiation and metastasis of NSCLC [46, 47], making them attractive targets for cancer therapy. The second messenger molecule, cAMP, primarily regulates cellular metabolism by activating protein kinase A (PKA) to phosphorylate downstream target proteins (such as enzymes and transcription factors), and it also participates in immune signal transduction. Research has indicated that cAMP modulates NSCLC stemness via the PKA/CREB1 pathway [48]. Additionally, cAMP‐binding proteins promote malignant metastasis in lung cancer by regulating transcription coactivators [49], positioning them as immunotherapeutic targets. Furthermore, studies have also demonstrated that the activation of the PI3K/Akt signaling pathway plays a crucial role in NSCLC growth, proliferation, angiogenesis, invasion, and metastasis. For instance, sustained activation of the PI3K/Akt pathway is closely associated with epithelial–mesenchymal transition (EMT) in NSCLC, promoting tumor invasion and metastasis [50]. PI3K/Akt regulates VEGF and hypoxia‐inducible factor 1 (HIF‐1) expression, thereby promoting tumor angiogenesis [51]. However, the downstream mechanisms through which the RPARP‐AS1/miR‐10b‐5p axis regulates NSCLC progression remain to be validated in further studies.

This study found that RPARP‐AS1 was downregulated in NSCLC and significantly correlated with poor patient prognosis, revealing its potential value as a liquid biopsy biomarker for NSCLC. Besides, RPARP‐AS1 overexpression inhibited tumor cell function, confirming the role of RPARP‐AS1 as a molecular sponge for miR‐10b‐5p, blocking its regulation of downstream oncogenes and thereby suppressing tumor progression. Previous studies have indicated that RPARP‐AS1 might inhibit lung cancer development by affecting endoplasmic reticulum stress or the immune microenvironment [18, 40], supporting this hypothesis. Therefore, subsequent studies could develop gene therapy vectors containing RPARP‐AS1 to control NSCLC [52]. For instance, delivering RPARP‐AS1 overexpression via adeno‐associated virus (AAV) or lipid nanoparticle (LNP) delivery systems could provide novel gene therapy strategies for NSCLC. Furthermore, this study demonstrated that miR‐10b‐5p was highly expressed in NSCLC, leading to a poorer patient prognosis. Combined with the regulatory effects of miR‐10b‐5p mimetic transfection on NSCLC cells, it was hereby hypothesized that its pro‐cancer mechanisms could include promoting tumor invasion by enhancing cell migration through regulating cytoskeletal rearrangement or EMT‐related genes (e.g., CRK and ITGB8) on the one hand, as well as participating in immune evasion on the other. Research has also indicated that miR‐10b‐5p may suppress immune checkpoint ligands (e.g., PD‐L1), thereby diminishing immunotherapy responses. Consequently, future studies should explore developing miRNA inhibitors [53]. This includes designing antisense oligonucleotides (ASOs) or small‐molecule inhibitors (e.g., Targaprimir‐10b) targeting miR‐10b‐5p to inhibit tumor progression. Additionally, combination therapy strategies—pairing miR‐10b‐5p inhibitors with immune checkpoint inhibitors (e.g., PD‐1 antibodies) or chemotherapeutic agents—may enhance antitumor efficacy [54].

However, several limitations of this study still warrant thorough discussion. First, the homogeneity of the sample size may introduce subtle biases in the results and affect their generalizability. In subsequent studies, multicenter experiments should be actively conducted to enhance the representativeness and reliability of the findings. Additionally, stratified analysis, particularly subgroup analyses (e.g., based on different pathological types or TNM staging), should be carried out to further examine the expression patterns of the target genes across various populations. Second, in vitro cell experiments cannot fully replicate the complex in vivo microenvironment, potentially overestimating or underestimating certain biological effects. Future work should therefore validate in vitro findings using in vivo animal models (e.g., mouse xenograft models) to more accurately assess the role of the RPARP‐AS1/miR‐10b‐5p axis in tumor progression. Third, the signaling pathways and underlying mechanisms of RPARP‐AS1/miR‐10b‐5p downstream target genes remain to be thoroughly validated. Despite the application of multiple databases for predicting miR‐10b‐5p downstream targets, bioinformatics predictions require experimental confirmation. Subsequent studies should integrate high‐throughput sequencing technologies (e.g., RNA‐seq and miRNA‐seq) with proteomics analysis to comprehensively screen and validate miR‐10b‐5p target genes, thereby elucidating potential mechanisms involving related signaling pathways (e.g., PI3K‐Akt) in NSCLC progression. Fourth, due to limitations in experimental conditions and technical platforms, a fluorescence in situ hybridization (FISH) assay enabling direct visualization of the subcellular localization of RPARP‐AS1 was not conducted in the current study. While the cytoplasmic distribution of RPARP‐AS1 was clarified by combining a nuclear‐cytoplasmic fractionation assay with bioinformatics prediction, further intuitive verification via FISH should be performed in the follow‐up research to consolidate the localization evidence.

## 5. Conclusion

In summary, this study identified the role of RPARP‐AS1 targeting miR‐10b‐5p in regulating NSCLC cell function. Specifically, overexpression of RPARP‐AS1 inhibited miR‐10b‐5p, thereby suppressing lung cancer cell proliferation, migration, and invasion and controlling NSCLC progression. Enrichment analysis of overlapping genes revealed that the target genes of miR‐10b‐5p primarily functioned in focal adhesion and axon development. However, the regulatory mechanism of the RPARP‐AS1/miR‐10b‐5p axis in NSCLC should still be further elucidated.

## Funding

This study was funded by the Medical Science Research Project of Hebei (Grant 20250351).

## Conflicts of Interest

The authors declare no conflicts of interest.

## Supporting Information

Additional supporting information can be found online in the Supporting Information section.

## Supporting information


**Supporting Information** Figure S1: Prediction of RPARP‐AS1 subcellular localization using two bioinformatics tools. (A) iLoc‐LncRNA 2.0 analysis shows that RPARP‐AS1 is localized in the cytoplasm (including cytosol) with a probability score of 0.705615. (B) LncLocator analysis shows that RPARP‐AS1 has the highest score in the cytosol (0.782), including predominant cytoplasmic distribution. Table S1: STR profiling and contamination monitoring results of the research cells.

## Data Availability

The data that support the findings of this study are available from the corresponding author upon reasonable request.
