# Supplementary material for: RPARP‐AS1 Targets miR‐10b‐5p to Influence Prognosis in Non‐Small Cell Lung Cancer and Inhibit Tumor Progression
Source: Anal Cell Pathol (Amst). 2026 Oct 6;2026:8817867. doi: 10.1155/ancp/8817867 (PMC13639925; doi:10.1155/ancp/8817867)
Supplement: Supplementary file 1 [file ANCP-2026-8817867-s001.zip › Supplementary Table 1.docx]

**Supplementary Table 1** Cell Short Tandem Repeat (STR) Profiling Information

| Cell Name | STR results | |
| --- | --- | --- |
| Beas-2B | Amelogenin: X, Y  CSF1PO: 9, 12  D13S317: 13  D16S539: 12  D5S818: 12,13 | D7S820: 10, 13  TH01: 7, 9.3  TPOX: 6, 11  vWA: 17, 18 |
| A549 | Amelogenin: X, Y  CSF1PO: 10,12  D13S317: 11  D16S539: 11,12  D5S818: 11 | D7S820: 8,11  THO1: 8,9.3  TPOX: 8,11  vWA: 14 |
| HCC1195 | Amelogenin：X, Y  CSF1PO：12  D13S317：12  D16S539：9  D5S818：13 | D7S820：10,11  THO1：7  TPOX：8,11  vWA：15,17 |
| H1975 | Amelogenin: X  CSF1PO: 12  D13S317: 10,13  D16S539: 9,12  D5S818: 11,12 | D7S820: 8,11  THO1: 7  TPOX: 8,11  vWA: 18 |
| NCI-H460 | Amelogenin: X, Y  CSF1PO: 11,12  D13S317: 13  D16S539: 9  D5S818: 9,10 | D7S820: 9,12  THO1: 9.3  TPOX: 8  vWA: 17 |

All STR results above were obtained from the cell bank of the Chinese Academy of Sciences Type Culture Collection.
